# Factors associated with functional health literacy and the quality of life of riverside residents served by the primary care network in the Brazilian amazon: a cross-sectional study

**DOI:** 10.1186/s12875-024-02684-y

**Published:** 2024-12-19

**Authors:** Ana Kedma Correa Pinheiro, Carlos Eduardo Raymundo, Eliene do Socorro da Silva Santos, Marcio Yrochy Saldanha dos Santos, Adriana de Oliveira Sarefino, Maria Helena do Nascimento Souza, Ingrid Bentes Lima, Raquel Gomes da Silva, Laura Maria Vidal Nogueira

**Affiliations:** 1 Escola de Enfermagem Anna Nery da Universidade Federal do Rio de Janeiro (UFRJ), Rio de Janeiro, Rio de Janeiro, Brazil; 2 Faculdade de Ciências Médicas da Universidade do Estado do Rio de Janeiro (UERJ), Rio de Janeiro, Rio de Janeiro, Brazil; 3 Escola de Enfermagem Magalhães Barata da Universidade do Estado do Pará (UEPA), Belém, Pará Brazil

**Keywords:** Health Literacy, Quality of Life, Socioeconomic Factors, Rural Population Health, Primary Health Care

## Abstract

**Background:**

the riverside population lives in a vulnerable social situation, shaped by geographical, economic, social, and educational aspects that have repercussions on health literacy, the limitations of which can compromise Quality of Life. These specificities influence the actions of Primary Health Care, especially in the rural context. This study aimed to assess the factors associated with Functional Health Literacy and Quality of Life among riverside residents of the Brazilian Amazon who use Primary Health Care.

**Methods:**

a cross-sectional study with 312 users of a riverside Family Health Team, using the Health Literacy Test, classified as adequate, limited, and inadequate, and the Study Short Form 12 Health Survey questionnaire, analyzing the physical and mental components in isolation. A theoretical model was built to assess the associations between sociodemographic and environmental variables and the Functional Health Literacy and Quality of Life outcomes. The Functional Health Literacy outcome was considered as two dichotomous variables (inadequate versus adequate; limited versus adequate), while the Quality of Life outcomes were considered as counts, with a Poisson distribution. Thus, a structural equation model was used to adjust the proposed theoretical model.

**Results:**

there was a worsening in inadequate Functional Health Literacy, associated with females, aged over 40, elementary school education, living close to the health service, and using only a cell phone for communication. Factors that compromised physical Quality of Life: livelihood problems; inadequate literacy; age range 40–59; and having two children or more. And those that worsened mental Quality of Life: age over 40; having a family allowance; and being in control of their medication.

**Conclusions:**

sociodemographic, environmental, and economic factors and adherence to medication by river communities have been shown to be associated with Health Literacy and Quality of Life. Knowing these implications is fundamental for health provision. These findings can support the formulation of strategies in health services to improve Health Literacy and Quality of Life.

## Background

Health Literacy (HL) describes the skills and abilities of users to engage with health information and services, to promote access, understanding, assessment, and use of health information and services, to promote and maintain good health and well-being for themselves and those around them [1, 2].

HL can be classified into three levels: (i) functional - communication of information using numerical and textual interpretation skills, (ii) interactive - development of personal skills, and (iii) critical - personal and community empowerment [1].

Thus, oral and written communication associated with health services determines how health information is received and favors the implementation of self-care, in which the level of FHL implies health promotion and prevention actions and clinical outcomes, which has repercussions on Quality of Life (QoL) [3].

In this sense, this study opted for the conceptions of the theoretical model of HL [4], which considers knowledge, motivation, and skills for maintaining or improving QoL.

QoL consists of a person’s perception of their position in life [5], understood as a level of well-being that is essential for living well [6]. A survey by the Brazilian Institute of Geography and Statistics (*Instituto Brasileiro de Geografia e Estatística* - IBGE) revealed that the Multidimensional Index for Loss of Quality of Life (*Índice Multidimensional para a Perda de Qualidade de Vida* - IPQV) was more pronounced in rural areas, among black or brown people, women, those with no schooling and residents of the North and Northeast of Brazil [7].

The state of Pará, located in the north of Brazil, has around 50% of its municipalities with a rural riverside population, living on the banks of the Amazonian rivers and depending almost exclusively on the Unified Health System (UHS) [8]. In Brazil, health care for river dwellers is based on the National Primary Care Policy (PNAB) and the National Policy for the Comprehensive Health of Rural, Forest, and Water Populations (PNSIPCFA), which includes multi-professional teams and modalities of river and riverine Family Health Teams (FHT) [9, 10].

Although these policies have weaknesses and limitations in their full implementation, overcoming the problems of daily life on the river is in line with achieving the Sustainable Development Goals (SDGs) for 2030 [11] and constitutes parameters for negotiating and defining climate policies at the 30th Conference of the Parties (COP 30) of the United Nations Framework Convention on Climate Change (UNFCCC), which is based in the Brazilian Amazon.

Even though the scientific literature shows the importance of assessing the living conditions of the riverside population [8, 12, 13], in Brazil there is still a gap in knowledge about the health situation of people living in rural areas, which reinforces the need for more in-depth studies on the reality of Amazonian populations, given that, in a search carried out in seven databases, covering HL and QoL, four publications were identified that had been developed with rural populations [14–17] and none with riverside populations.

Such studies were conducted in China and showed that low HL leads to a negative perception of QoL in women [14], women with chronic diseases [15], elderly people living with diabetes [16], and people with hypertension [17].

Thus, it is understood that people with greater limitations in FHL tend not to understand their health status, as well as have limitations in performing behaviors that favor disease control, good self-management in health, and the adoption of healthy behavioral habits that lead to a better QoL [18].

According to a literature review, there are no studies on the Brazilian reality that have evaluated the relationship between HL and QoL in riverside populations. Likewise, there are no national studies that cover LS associated with QoL linked to the characteristic factors of the environment in which these population groups in the Amazon live.

This study aimed to assess the factors associated with FHL and QoL among riverine dwellers in the Brazilian Amazon who are Primary Health Care (PHC) users. With this study, we hope to contribute to the indication of evidence related to HL and QoL in the context of traditional populations in the Amazon, who experience situations of social vulnerability.

## Methods

### Study design and location

**Fig. 1:**
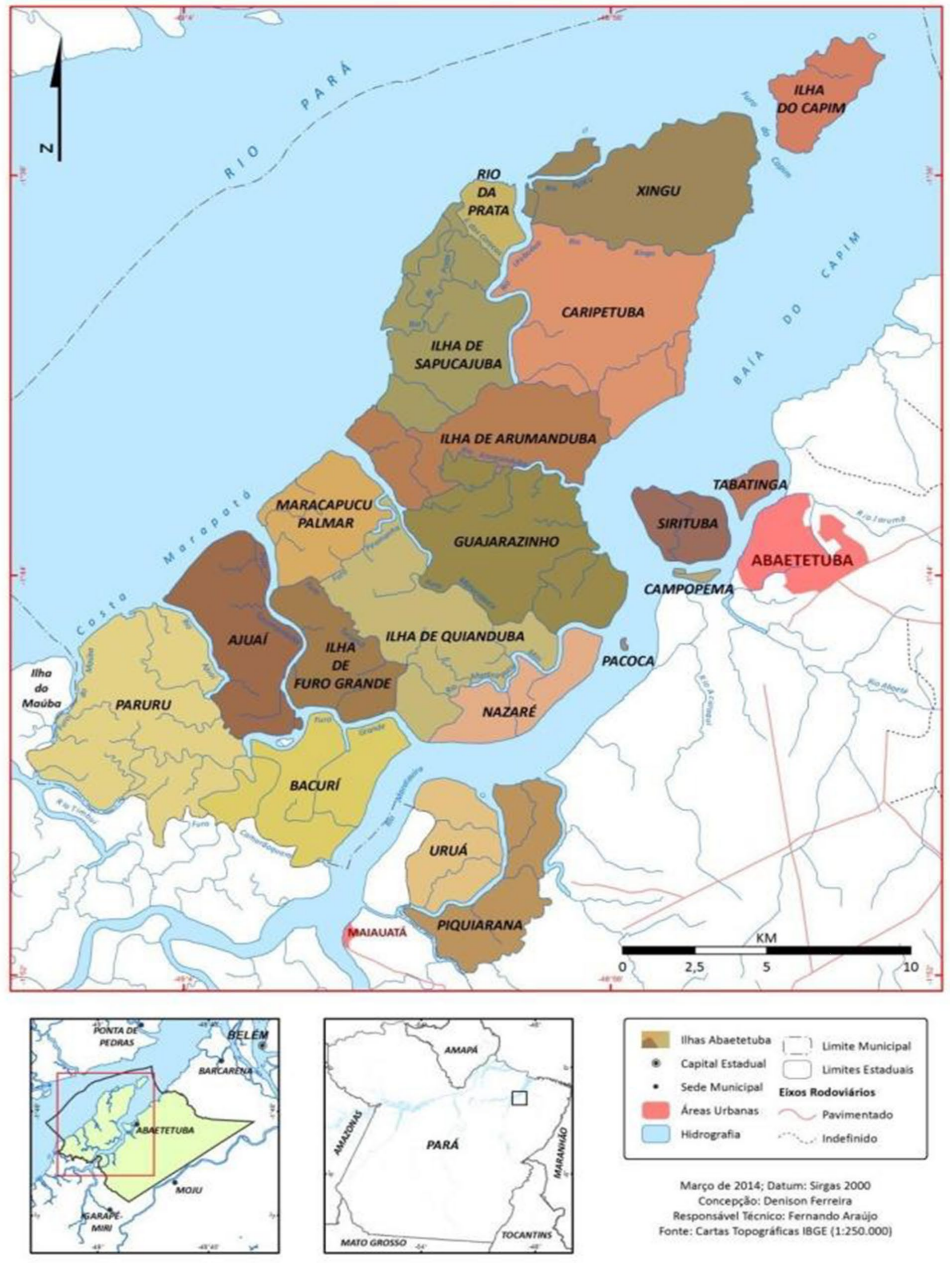
Spatial location of the study area in Abaetetuba (PA), Brazil

This is a cross-sectional study, based on the Strengthening the Reporting of Observational Studies in Epidemiology [19], carried out in a riverside area of a municipality in the rural area of Abaetetuba (PA), in the Brazilian Amazon (Fig. 1).

### Participants

The population was made up of users linked to the Riverside Family Health Team (RFHT) located on the river called Arumanduba, which serves four territories with eight micro-areas, two of which are not covered by Community Health Agents (CHA), and is the only riverside unit in the municipality. The team includes a nurse, a doctor, three nursing technicians, a dental surgeon, an oral health assistant, and 14 CHAs [20].

The inclusion criteria were: users aged ≥ 18 years; of both sexes; who could read and write; residents of the region, and who used the RFHT service. The exclusion criteria were users with visual or hearing limitations and cognitive or behavioral impairment, recorded in medical records.

The sample size was calculated using the Cochran technique, considering a 5% sampling error, follow the formula [21]: $\:n=\frac{N}{1+(N-1){\epsilon\:}^{2}}$. Where: N = population size; n = sample size; ε = 0,05 (error).

Thus, the sample calculation for the RFHT population resulted in a total of 304 individuals, with 312 participants included in the study. The RFHT population corresponds to 1,262 users aged ≥ 18 years, as established in the registry in the Territorialization System of the Unified Health System (e-SUS Território).

This study has convenience sampling, by quotas of participants, including equitable representation from each of the four territories [22], as well as considering the different strata of education, according to the criteria pre-established by the IBGE [23].

### Data collection instrument

To assess Functional Health Literacy (FHL), the most widely used instrument in the literature is the Test of Functional Health Literacy in Adults (TOFHLA). In this study, we used the Health Literacy Test (TLS) built using the TOFHLA, which makes it possible to assess the numerical ability and reading comprehension of Brazilians [24].

To obtain the data, we used the TLS, which has an internal consistency of 0.953 (Cronbach’s alpha) and makes it possible to assess two domains of literacy [24]. To assess QoL, we used the 12-item Short Form Health Survey (SF-12), version 1.0, created in 1994 [25], validated in Brazil [26], and then evaluated with good psychometric properties, with a Cronbach’s alpha of 0.836 [27].

To trace the socio-demographic profile and livelihood problems, we used the questionnaire drawn up by the research center, the National Confederation of Agricultural Workers [28]. The instrument consists of two parts, only the first of which was used, with 12 questions relating to profile/lifestyle and six relating to production/work in the field.

### Data collection

This was preceded by a pilot study with river residents in other areas of the municipality, which allowed for adjustments in the application of the instruments and in the direction of the research team and the fieldwork. Training was then given to five interviewers, undergraduate nursing students from a public university, who had taken the subject Nursing and the Traditional Populations of the Amazon.

Data collection took place at the RFHT’s facilities, while users were waiting for care, and at home during scheduled visits by the CHWs, at the participants’ discretion, between February and May 2021.

### Data analysis

The data was double-entered into a database created in the Statistical Package for the Social Sciences (SPSS) version 22.0.

The TLS score followed the authors’ recommendations [24, 29, 30], in which one was given for each correct answer and zero for incorrect answers and/or self-declaration of not knowing and/or not filling in. The numerical part contains 17 items, which were multiplied by 2.941 to convert to a score from 0 to 50 points, following the recommendations of the original instrument [30]. The reading comprehension part was not weighted. The scores were obtained from the sum of the domains, considering the score from zero to 100. Scores between 75 and 100 were classified as adequate literacy, 60 to 74 as limited literacy, and zero to 59 as inadequate literacy [24, 30].

The SF-12 score followed the recommendations of the original instrument [25], with a graduated Likert-type scale, whose score varies according to the composition of the question, ranging from zero to 100, in which the highest scores are associated with the best levels of QoL. A total score was not established and each component was analyzed in isolation, grouped into Physical Component Summary (PCS) and Mental Component Summary. (MCS).

To define the component scores, the algorithm used to calculate the SF-12 score was taken into account, which has the 1998 US population average as a parameter, adopted in Brazilian and international studies [25–27, 31].

Structural Equation Modeling (SEM) was used to assess the association between sociodemographic, economic, and environmental characteristics and HL and QoL. This approach was considered in order to estimate the total, direct, and indirect effects of inadequate and limited literacy, as well as Socioeconomic Status (SES), concerning physical and mental QoL scales.

**Fig. 2:**
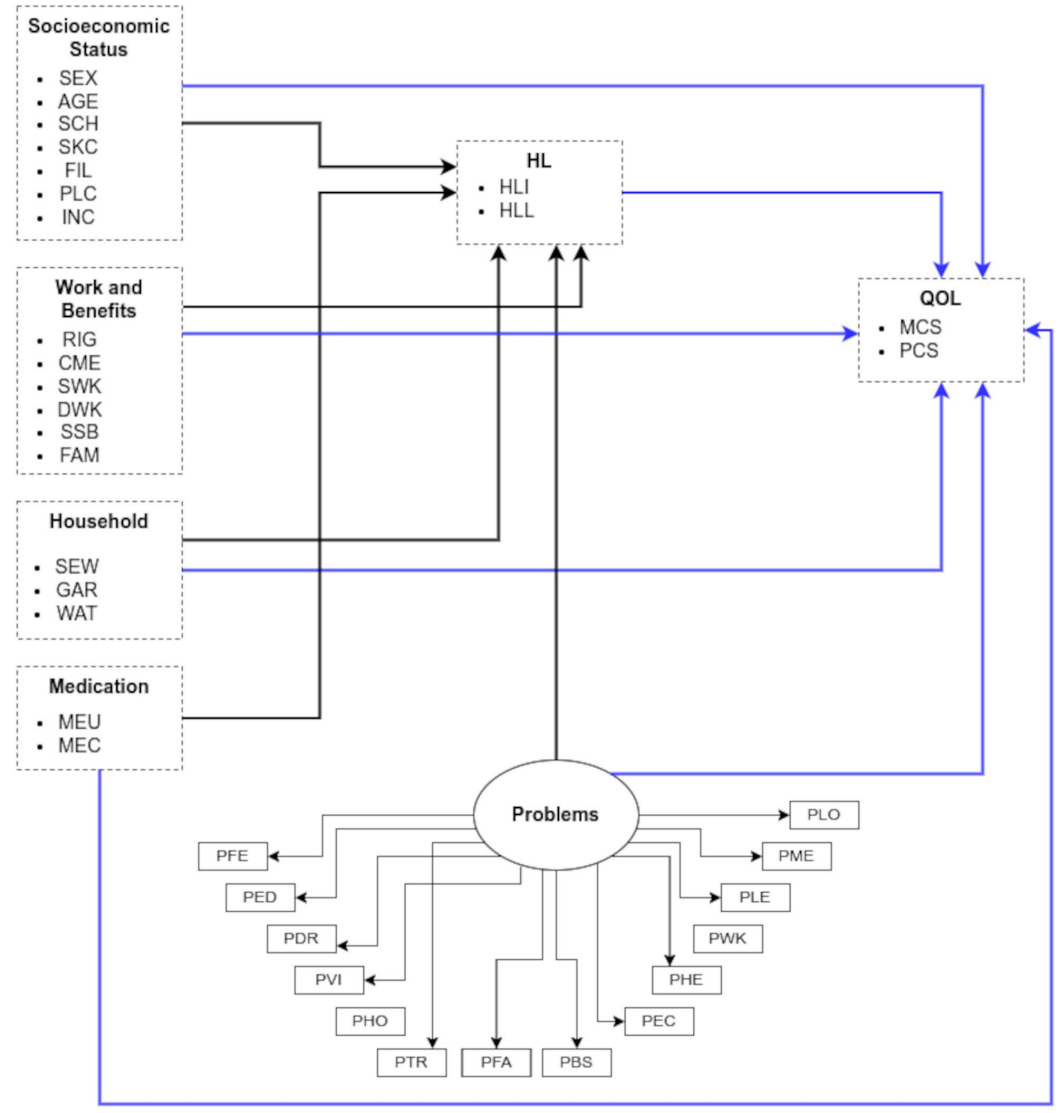
Theoretical model health literacy. Note: SEX: Sex; AGE: Age group; SCH: Schooling; SKC: Skin color/race; FIL: Number of children; PLC: Proximity to health service; INC: Income; RIG: Defense of rights; CME: Means of communication; SWK: Start of work activities; DWK: Daily working hours; SSB: Social Security benefit; FAM: Family Allowance (*Bolsa Família*); SEW: Sanitary sewage; GAR: Destination of garbage; WAT: Water supply; MEU: Medication use; MEC: Medication control; PFE: Problems with feeding; PED: Problems with education; PDR: Problems with drugs; PVI: Problems with violence; PHO: Problems with housing; PTR: Problems with transportation: PFA: Family problems; PBS: Basic sanitation problems; PEC: Economic problems; PHE: Health problems; PWK: Work problems; PLE: Leisure problems; PME: Media problems; PLO: Loneliness problems; HLI: Inadequate FHL; HLL: Limited FHL; MCS: Mental Component Summary; PCS: Physical Component Summary

The Sørensen model [4] includes three determinant groups at the individual level (socioenvironmental, personal, and situational) and four determinant groups populations (health service use, health behavior, participation, and equity). Thus, an adaptation was made of the factors associated with HL and QoL, grouped into the following blocks: socio-economic status; work and benefits; household condition, use, and control of medication (Fig. 2).

In the model by Sørensen et al. [4], the socio-environmental determinants were adapted in the household block, containing the following variables: sewage disposal (SEW), water supply (WAT), and waste disposal (GAR). The personal and situational determinants were adapted in the socioeconomic status block, which included the variables: sex (SEX), age group (AGE), education (SCH), color/race (SKC), number of children (FIL), proximity to health service (PLC) and income (INC), in addition to including the variables of the household condition block, which consists of sewage disposal (SEW), waste disposal (GAR) and water supply (WAT).

The determinants related to the use of health services were adapted to the use of medication, which involves medication use (MEU) and medication control (MEC). The problems assumed by the riverside population as health-related behaviors were considered, called individual subsistence problems, which include food problems (PFE), education problems (PED), drug problems (PDR), violence problems (PVI), housing problems (PHO), transportation problems (PTR), family problems (PFA), sanitation problems (PBS), economic problems (PEC), health problems (PHE), work problems (PWK), leisure problems (PLE), media problems (PME) and loneliness problems (PLO).

Although these problems comprise contextual and individual levels, which are different from the original model, in this study they were included together. According to the authors, improving the FHL process could lead to an improvement in the health outcome of QoL (Fig. 2).

Figure 2 was constructed based on the associations conjectured by the author and adapted in this study. The smaller rectangles represent the variables (indicators) that were measured in the questionnaire. The items (bullets) inserted within the dashed rectangles were also considered variables. These rectangles represent groups of variables to facilitate visualization of the theoretical model. The black lines with arrows (paths) represent the associations of the independent variables for the FHL, while the blue lines represent the associations of all the indicators for QoL. The circle characterized by “individual problems” represents a latent variable (factor), i.e. one that was not measured in the questionnaire. The “individual problems” factor also has an association with FHL and another with QoL.

The FHL outcome was considered as a dichotomous variable according to the following cut-off points: (i) inadequate, zero to 59; (ii) limited, 60 to 74 points. Logistic regressions were then evaluated for FHL outcomes. Finally, the QoL outcomes were considered as counts, and evaluated as a Poisson regression. Although the variables were presented in blocks, they were analyzed in a complete multivariate model, considering the backward strategy to end up with a significant model.

The descriptive analyses were carried out in the R statistical package version 4.1.1, while the SEM model was implemented in the MPlus software [32]. A value of *p* ≤ 0.05 was considered.

### Ethical considerations

The study complied with Resolution 466/2012 and 580/18 - CNS-MS and was approved by the Research Ethics Committee of the Undergraduate Nursing Course at the State University of Pará, under opinion 4.517.829 of February 1, 2021.

## Results

### Characteristics of the participants

Of the total (*n* = 312), 53 had adequate FHL, 54 limited, and 205 inadequate, with a mean age of 35.0 (SD 13.4 years) ranging from 18 to 78 years. The majority were female (*n* = 209; 67.0%), self-declared non-white (*n* = 285; 91.3%), had two or more children (*n* = 181; 58.8%), did not participate in advocacy spaces (*n* = 192; 61.7%), used a rural telephone (*n* = 192; 62.3%), had inadequate sanitary sewage (*n* = 181; 58.0%), adequate water supply (*n* = 237; 76.0%) and disposed of waste inadequately (*n* = 295; 95.2%) (Table 1).

**Table 1:** Functional Health Literacy and the sociodemog

Characteristics	Functional Health Literacy				
	Adequate *N* = 53	Limited *N* = 54	Inadequate *N* = 205	Total, *N* = 312	*p*-value^1^
**Sex**					< 0.001
Female	45 (84.9%)	42 (77.8%)	122 (59.5%)	209 (67.0%)	
Male	8 (15.1%)	12 (22.2%)	83 (40.5%)	103 (33.0%)	
**Age**					< 0.001^2^
Median (IQR)	28.0 (23.0–33.0)	28.5 (22.8–33.3)	35.0 (27.0–45.0)	32.0 (25.0–42.0)	
Range	18.0–54.0	18.0–70.0	18.0–78.0	18.0–78.0	
Mean (Standard Deviation)	28.8 (8.1)	29.9 (10.6)	38.0 (14.2)	35.0 (13.4)	
No information	0	2	2	4	
**Age group**					
18–29	30 (56.6%)	30 (57.7%)	62 (30.5%)	122 (39.6%)	
30–39	19 (35.8%)	17 (32.7%)	65 (32.0%)	101 (32.8%)	
40–49	2 (3.8%)	2 (3.8%)	33 (16.3%)	37 (12.0%)	
50–59	2 (3.8%)	1 (1.9%)	21 (10.3%)	24 (7.8%)	
60 and over	0 (0.0%)	2 (3.8%)	22 (10.8%)	24 (7.8%)	
No information	0	2	2	4	
**Skin color/race**					0.667
White	4 (7.5%)	3 (5.6%)	20 (9.8%)	27 (8.7%)	
Not white	49 (92.5%)	51 (94.4%)	185 (90.2%)	285 (91.3%)	
**Proximity to health service**					< 0.001
Close	30 (56.6%)	24 (44.4%)	57 (27.8%)	111 (35.6%)	
Far	23 (43.4%)	30 (55.6%)	148 (72.2%)	201 (64.4%)	
**Schooling**					< 0.001
Up to elementary school	15 (28.3%)	14 (25.9%)	165 (80.5%)	194 (62.2%)	
High school and college	38 (71.7%)	40 (74.1%)	40 (19.5%)	118 (37.8%)	
**Number of children**					< 0.001
Up to 1 child	25 (47.2%)	35 (67.3%)	67 (33.0%)	127 (41.2%)	
2 or more children	28 (52.8%)	17 (32.7%)	136 (67.0%)	181 (58.8%)	
No information	0	2	2	4	
**Defense of rights**					0.604
No	34 (64.2%)	36 (66.7%)	122 (59.8%)	192 (61.7%)	
Yes	19 (35.8%)	18 (33.3%)	82 (40.2%)	119 (38.3%)	
No information	0	0	1	1	
**Means of communication**					< 0.001
Cell phone with internet	33 (62.3%)	29 (53.7%)	51 (25.4%)	113 (36.7%)	
Rural phone	20 (37.7%)	25 (46.3%)	147 (73.1%)	192 (62.3%)	
None	0 (0.0%)	0 (0.0%)	3 (1.5%)	3 (1.0%)	
No information	0	0	4	4	
**Sanitary sewage**					0.669
Adequate	20 (37.7%)	25 (46.3%)	86 (42.0%)	131 (42.0%)	
Inadequate	33 (62.3%)	29 (53.7%)	119 (58.0%)	181 (58.0%)	
**Water supply**					0.193
Adequate	45 (84.9%)	42 (77.8%)	150 (73.2%)	237 (76.0%)	
Inadequate	8 (15.1%)	12 (22.2%)	55 (26.8%)	75 (24.0%)	
**Waste disposal**					0.200
Adequate	3 (5.7%)	0 (0.0%)	12 (5.9%)	15 (4.8%)	
Inadequate	50 (94.3%)	53 (100.0%)	192 (94.1%)	295 (95.2%)	
No information	0	1	1	2	
**Start of employment (in years)**					< 0.001^2^
Median (IQR)	15.0 (10.5–18.0)	14.0 (10.0–18.0)	12.0 (8.5–14.0)	12.0 (10.0–15.0)	
Range	7.0–26.0	6.0–28.0	3.0–31.0	3.0–31.0	
Mean (Standard Deviation)	15.2 (5.2)	14.4 (4.9)	11.6 (3.9)	12.5 (4.5)	
Never worked	18	13	26	57	
**Category in which work began**					< 0.001
Under 18	21 (60.0%)	29 (70.7%)	166 (92.7%)	216 (84.7%)	
18 to 31 years old	14 (40.0%)	12 (29.3%)	13 (7.3%)	39 (15.3%)	
Never worked	18	13	26	57	
**Daily working hours**					0.282
Up to 8 h	50 (94.3%)	50 (92.6%)	180 (87.8%)	280 (89.7%)	
More than 8 h	3 (5.7%)	4 (7.4%)	25 (12.2%)	32 (10.3%)	
**Social security benefit**					0.002
Yes	9 (17.0%)	9 (16.7%)	73 (35.6%)	91 (29.2%)	
No	44 (83.0%)	45 (83.3%)	132 (64.4%)	221 (70.8%)	
**Family Allowance**					0.593
Yes	32 (60.4%)	36 (66.7%)	139 (67.8%)	207 (66.3%)	
No	21 (39.6%)	18 (33.3%)	66 (32.2%)	105 (33.7%)	
**Income**					0.500
Up to 1 minimum wage	29 (54.7%)	34 (64.2%)	128 (63.1%)	191 (61.8%)	
More than 1 minimum wage	24 (45.3%)	19 (35.8%)	75 (36.9%)	118 (38.2%)	
No information	0	1	2	3	
**Problems with feeding**					0.759
No	44 (83.0%)	47 (87.0%)	178 (86.8%)	269 (86.2%)	
Yes	9 (17.0%)	7 (13.0%)	27 (13.2%)	43 (13.8%)	
**Problems with education**					0.101
No	36 (67.9%)	40 (74.1%)	166 (81.0%)	242 (77.6%)	
Yes	17 (32.1%)	14 (25.9%)	39 (19.0%)	70 (22.4%)	
**Problems with drugs**					0.722
No	47 (88.7%)	45 (83.3%)	177 (86.3%)	269 (86.2%)	
Yes	6 (11.3%)	9 (16.7%)	28 (13.7%)	43 (13.8%)	
**Problems with violence**					0.421
No	32 (60.4%)	38 (70.4%)	125 (61.0%)	195 (62.5%)	
Yes	21 (39.6%)	16 (29.6%)	80 (39.0%)	117 (37.5%)	
**Problems with housing**					0.007
No	48 (90.6%)	49 (90.7%)	201 (98.0%)	298 (95.5%)	
Yes	5 (9.4%)	5 (9.3%)	4 (2.0%)	14 (4.5%)	
**Problems with transportation**					0.104
No	47 (88.7%)	53 (98.1%)	182 (88.8%)	282 (90.4%)	
Yes	6 (11.3%)	1 (1.9%)	23 (11.2%)	30 (9.6%)	
**Family problems**					0.202
No	44 (83.0%)	47 (87.0%)	187 (91.2%)	278 (89.1%)	
Yes	9 (17.0%)	7 (13.0%)	18 (8.8%)	34 (10.9%)	
**Basic sanitation problems**					0.008
No	30 (56.6%)	36 (66.7%)	158 (77.1%)	224 (71.8%)	
Yes	23 (43.4%)	18 (33.3%)	47 (22.9%)	88 (28.2%)	
**Economic problems**					0.658
No	41 (77.4%)	44 (81.5%)	155 (75.6%)	240 (76.9%)	
Yes	12 (22.6%)	10 (18.5%)	50 (24.4%)	72 (23.1%)	
**Health problems**					0.486
No	26 (49.1%)	31 (57.4%)	99 (48.3%)	156 (50.0%)	
Yes	27 (50.9%)	23 (42.6%)	106 (51.7%)	156 (50.0%)	
**Problems with work**					0.662
No	41 (77.4%)	39 (72.2%)	160 (78.0%)	240 (76.9%)	
Yes	12 (22.6%)	15 (27.8%)	45 (22.0%)	72 (23.1%)	
**Problems with leisure**					> 0.999
No	52 (98.1%)	53 (98.1%)	201 (98.0%)	306 (98.1%)	
Yes	1 (1.9%)	1 (1.9%)	4 (2.0%)	6 (1.9%)	
**Problems with means of communication**					0.087
No	45 (84.9%)	50 (92.6%)	193 (94.1%)	288 (92.3%)	
Yes	8 (15.1%)	4 (7.4%)	12 (5.9%)	24 (7.7%)	
**Problems with loneliness**					> 0.999
No	52 (98.1%)	53 (98.1%)	198 (96.6%)	303 (97.1%)	
Yes	1 (1.9%)	1 (1.9%)	7 (3.4%)	9 (2.9%)	
**Use of medication**					0.325
Correct	43 (81.1%)	37 (68.5%)	152 (74.1%)	232 (74.4%)	
Incorrect	10 (18.9%)	17 (31.5%)	53 (25.9%)	80 (25.6%)	
**Control of medication**					0.622
Correct	34 (64.2%)	32 (59.3%)	136 (66.3%)	202 (64.7%)	
Incorrect	19 (35.8%)	22 (40.7%)	69 (33.7%)	110 (35.3%)	
**PCS score**					0.090^2^
Median (IQR)	46.5 (38.6–51.0)	48.5 (38.7–52.5)	43.5 (34.8–51.9)	44.7 (36.4–52.1)	
Range	20.9–58.7	20.4–57.7	14.1–62.1	14.1–62.1	
Mean (Standard Deviation)	44.9 (8.4)	45.7 (9.2)	42.5 (10.7)	43.5 (10.2)	
**MCS score**					0.776^2^
Median (IQR)	49.2 (41.4–53.8)	51.7 (40.6–55.2)	49.8 (40.4–55.3)	50.2 (40.6–55.1)	
Range	23.4–64.3	19.3–67.0	20.6–66.0	19.3–67.0	
Mean (Standard Deviation)	47.3 (9.2)	48.2 (10.5)	47.7 (9.5)	47.8 (9.6)	

raphic, economic and environmental characteristics of the riverside residents (*n* = 312)

Note: IQR: Interquartile Range; PCS: Physical Component Summary; MCS: Mental Component Summary

^1^Pearson’s Chi-squared test

^2^Kruskal-Wallis test

The riverside residents started working at an average age of 12.5 (SD 4.5 years), ranging from three to 31 years, mostly under the age of 18 (*n* = 216; 84.7%), worked up to eight hours a day (*n* = 280; 89.7%), were not social security beneficiaries (*n* = 221; 70.8%), received a family allowance (*n* = 207; 66.3%), and had an income of up to one minimum wage (*n* = 191; 61.8%) (Table 1).

The most common problems experienced in daily life were: health (*n* = 156; 50.0%); violence (*n* = 117; 37.5%); and basic sanitation (*n* = 88; 28.2%). In addition, the majority of river dwellers made correct use (*n* = 232; 74.4%) and control (*n* = 202; 64.7%) of medicines (Table 1).

According to Table 1, the average PCS QoL score was 43.5 (SD 10.2), ranging from 14.1 to 62.1. The mean MCS score was 47.8 (SD 9.6), ranging from 19.3 to 67.0.

### Bivariate analysis

The statistical analysis associated with sociodemographic and environmental factors, medication use and control and FHL are presented in Table 1. The results indicated that ten factors significantly influenced the level of FHL, namely: sex (*p* < 0.001); age (*p* < 0.001); proximity to the health service (*p* < 0.001); schooling (*p* < 0.001); number of children (*p* < 0.001); means of communication (*p* < 0.001); initial age of working activities (*p* < 0.001); social security benefit (*p* = 0.002); problems with housing (*p* = 0.007); and, basic sanitation (*p* = 0.008) (Table 1).

Table 2 shows the association between QoL and sociodemographic, environmental, medication use and control, problematic, and FHL factors. The factors that showed an association with physical QoL were: age group (*p* < 0.001); schooling (*p* = 0.039); number of children (*p* < 0.001); participation in legal defense (*p* = 0.029); means of communication (*p* = 0.005); waste disposal (*p* = 0.041); social security benefits (*p* = 0.004); problems with feeding (*p* = 0.004), education (*p* = 0.025), transportation (*p* = 0.052), financial (*p* = 0.054), and health (*p* = 0.014). The transport and financial problems variables were not significant but were maintained in the final model for parsimony reasons and to improve the final fit of the model.

**Table 2:** Quality of life and the socio-demographic, economic and environmental characteristics of the riverside residents (*n* = 312)

Characteristics	PCS score		MCS score	
	*N* = 312^1^	*p*-value^2^	*N* = 312^1^	*p*-value^2^
**Sex**		0.813		0.049
Female	45 (36–52)		48 (40–55)	
Male	44 (36–51)		52 (43–56)	
**Age group**		< 0.001		0.180
18–29	47 (40–54)		52 (42–55)	
30–39	46 (38–52)		50 (41–55)	
40–49	40 (32–47)		44 (40–53)	
50–59	33 (28–42)		50 (46–58)	
60 and over	38 (35–46)		44 (35–55)	
No information	4		4	
**Skin color/race**		0.637		0.618
White	43 (36–52)		51 (43–56)	
Non-white	45 (36–52)		50 (41–55)	
**Proximity to health service**		0.825		0.623
Nearby	46 (37–52)		51 (42–55)	
Far	44 (36–52)		50 (41–55)	
**Schooling**		0.039		0.283
Up to elementary school	44 (35–52)		48 (40–55)	
High school and college	47 (39–53)		52 (42–55)	
**Number of children**		< 0.001		0.422
Up to 1 child	47 (40–53)		52 (42–55)	
2 or more children	41 (34–52)		49 (41–55)	
No information	4		4	
**Defense of rights**		0.029		0.929
No	46 (37–53)		51 (40–55)	
Yes	43 (34–51)		49 (41–55)	
No information	1		1	
**Means of communication**		0.005		0.569
Cell phone with internet	47 (39–53)		50 (42–55)	
Rural phone	42 (35–52)		51 (41–56)	
None	52 (51–56)		42 (38–47)	
No information	4		4	
**Sanitary sewage**		0.077		0.269
Adequate	46 (38–52)		51 (43–55)	
Inadequate	44 (35–52)		49 (40–55)	
**Water supply**		> 0.999		0.857
Adequate	45 (36–52)		50 (41–55)	
Inadequate	45 (36–52)		51 (40–55)	
**Waste disposal**		0.041		0.270
Adequate	50 (45–53)		52 (47–56)	
Inadequate	44 (36–52)		50 (40–55)	
No information	2		2	
**Start of work activities**		0.062		0.214
Under 18	42 (34–52)		50 (40–55)	
18 to 31 years old	47 (40–52)		52 (45–55)	
Never worked	57		57	
**Daily working hours**		0.745		0.387
More than 8 h	43 (37–51)		52 (43–56)	
Up to 8 h	45 (36–52)		50 (41–55)	
**Social security benefit**		0.004		0.630
Yes	41 (34–51)		49 (41–55)	
No	46 (38–53)		50 (41–55)	
**Family Allowance**		0.123		0.074
Yes	46 (37–53)		48 (40–55)	
No	43 (36–51)		52 (42–56)	
**Income**		0.448		0.312
Up to 1 minimum wage	45 (36–53)		49 (41–54)	
More than 1 minimum wage	45 (37–52)		51 (40–56)	
No information	3		3	
**Problems with food**		0.004		0.834
No	46 (37–52)		50 (41–55)	
Yes	38 (32–47)		51 (41–53)	
**Problems with education**		0.025		0.254
No	44 (35–52)		51 (41–55)	
Yes	46 (39–53)		48 (41–54)	
**Problems with drugs**		0.993		0.551
No	44 (37–52)		51 (41–55)	
Yes	46 (35–52)		50 (40–55)	
**Problems with violence**		0.586		0.560
No	45 (37–52)		49 (40–55)	
Yes	43 (35–52)		51 (41–56)	
**Problems with housing**		0.291		0.004
No	45 (36–52)		49 (40–55)	
Yes	38 (34–51)		55 (52–59)	
**Problems with transportation**		0.052		0.458
No	46 (37–52)		51 (40–55)	
Yes	39 (32–49)		47 (42–54)	
**Family problems**		0.721		0.256
No	45 (36–52)		51 (41–55)	
Yes	42 (37–51)		46 (38–53)	
**Problems with basic sanitation**		0.106		0.554
No	44 (35–52)		51 (41–55)	
Yes	46 (38–53)		48 (40–56)	
**Economic problems**		0.054		0.380
No	46 (37–52)		51 (41–55)	
Yes	41 (33–50)		48 (40–54)	
**Health problems**		0.014		0.004
No	47 (38–53)		51 (43–57)	
Yes	41 (35–51)		47 (40–54)	
**Problems with work**		0.573		0.422
No	45 (36–52)		49 (41–55)	
Yes	43 (36–53)		52 (41–56)	
**Problems with leisure**		0.676		0.309
No	45 (36–52)		50 (41–55)	
Yes	45 (43–52)		43 (41–47)	
**Problems with means of communication**		0.338		0.184
No	44 (36–52)		50 (41–55)	
Yes	48 (40–51)		45 (35–55)	
**Problems with loneliness**		0.916		0.016
No	45 (37–52)		51 (41–55)	
Yes	47 (34–54)		37 (31–48)	
**Use of medication**		0.459		0.494
Correct	45 (36–52)		50 (41–55)	
Incorrect	45 (38–53)		51 (42–55)	
**Control of medication**		0.883		0.077
Correct	45 (36–52)		51 (42–56)	
Incorrect	44 (37–52)		49 (37–54)	
**Functional Health Literacy**		0.090		0.776
Adequate	47 (39–51)		49 (41–54)	
Limited	48 (39–53)		52 (41–55)	
Inadequate	43 (35–52)		50 (40–55)	

Note: IQR: Interquartile Range; PCS: Physical Component Summary; MCS: Mental Component Summary. ^1^PCS and MCS score: Median (IQR). ^2^Wilcoxon rank sum test; Kruskal-Wallis test

With regard to mental QoL, an association was found with sex (*p* = 0.049) and problems with housing (*p* = 0.004), health (*p* = 0.004) and loneliness (*p* = 0.016).

### Structural equation modeling

Table 3 shows the results of the adjusted SEM model with the Odds Ratio (OR) estimates, the respective confidence intervals, and p-values. About limited literacy, no variable showed a statistically significant association. The variable inadequate waste disposal had a point estimate close to zero, which is why the result is not shown in the illustrations.

Living close to the health service, being female, being over 40 years old, having primary education, and only using a cell phone for communication are conditions that favor inadequate FHL. The exception was the factor related to “livelihood problems”, which showed a negative association with inadequate FHL.

Livelihood problems, inadequate literacy, the 40–49 and 50–59 age groups, and having two children or more are variables that worsen physical QoL. On the other hand, the variables female sex, primary education, and limited literacy improved physical QoL. Age over 40, having a family allowance, and being in control of their medication worsened mental QoL, while inadequate waste disposal was associated improved mental QoL.

The construct considering problems was constructed with the variables education, violence, housing, transportation, and basic sanitation. These problems were recognized as protective factors for inadequate literacy and physical QoL.

**Table 3:** Association of significant variables for the outcomes of inadequate literacy, unlimited, physical QoL, and mental QoL. Pará (Brazil) (*n* = 312)

Variable	Odds Ratio	95% CI	*p*-value
Inadequate literacy			
Problem fator	0.02	0–0.32	0.007
Proximity to health service: Nearby	7.78	1.34–45.29	0.022
Sex: Female	16.28	2.1–126.49	0.008
Age group: 40–49 years	48.47	1.77–1325.41	0.021
Age group: 50–59	29.14	1.29–660.05	0.034
Age group: 60 and over	202.15	1.28 – Inf	0.040
Schooling: Elementary	300.97	8–11327.18	0.002
Means of communication: Mobile phone	7.48	1.03–54.14	0.046
Physical QoL			
Problem fator	0.81	0.77–0.84	< 0.001
Literacy: Limited	1.27	1.15–1.4	< 0.001
Literacy: Inadequate	0.8	0.72–0.88	< 0.001
Sex: Female	1.06	1–1.13	0.045
Age range: 40–49 years	0.88	0.8–0.96	0.004
Age: 50–59	0.81	0.73–0.91	< 0.001
Schooling: Elementary	1.19	1.1–1.29	< 0.001
Number of children: two or more	0.94	0.89–0.99	0.02
Mental QoL			
Age group: 40–49	0.94	0.89–0.99	0.032
Age group: 60 and over	0.86	0.79–0.93	< 0.001
Bolsa Família: Yes	0.93	0.89–0.97	0.002
Garbage: Inadequate	1.1	1.01–1.2	0.023
Medication control: Yes	0.96	0.93–1	0.045

**Fig. 3:**
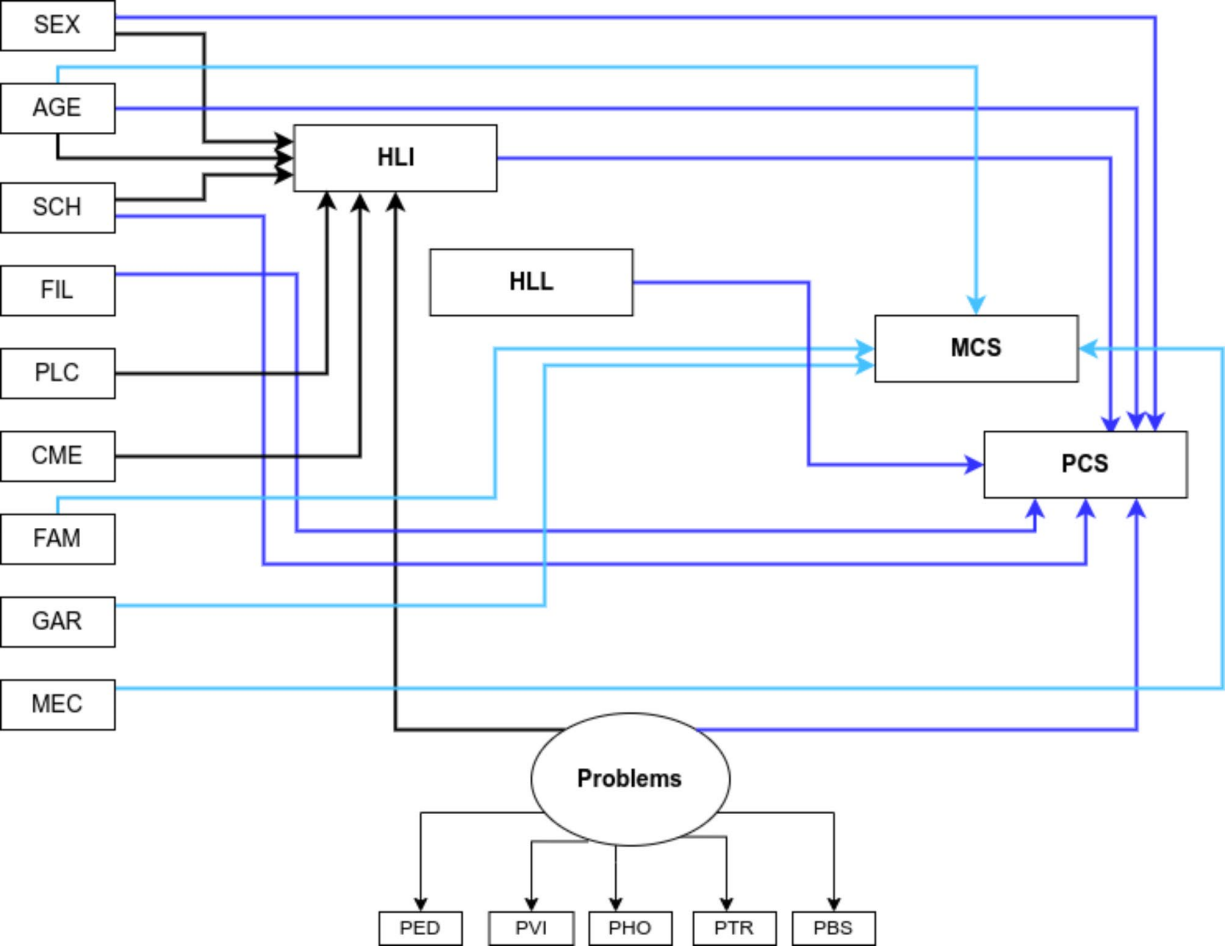
Final SEM diagram. Note: SEX: Sex; AGE: Age group; SCH: Schooling; FIL: Number of children; PLC: Proximity to health service; CME: Means of communication; FAM: Bolsa Família; GAR: Garbage disposal; MEC: Medication control; PED: Problems with education; PVI: Problems with violence; PHO: Problems with housing; PTR: Problems with transportation; PBS: Problems with basic sanitation; HLI: Inadequate FHL; HLL: Limited FHL; MCS: Mental Component Summary; PCS: Physical Component Summary

Figure 3 shows the SEM diagram in the final adjusted model. In the group of socio-economic variables, only the income variable (INC) did not remain in the adjustment. In the work and benefits block, means of communication (CME) and *Bolsa Família* (FAM) remained, while in the household group, only garbage disposal (GAR) remained. Among the variables related to medication, only medication control (MEC) remained in the final adjustment. All the blocks of variables had a significant association with HL and QoL, except for the medication group, which only had a path with mental QoL. After adjustment, the “individual problems” factor was made up of the variables problems with education (PED), violence (PVI), housing (PHO), transportation (PTR), and basic sanitation (PBS). Only the “individual problems” path associated with inadequate literacy (HLI) remained.

## Discussion

This study identified that socioeconomic characteristics were significantly associated with inadequate FHL. On the other hand, it was observed that FHL assumes an improved status when the existence of subsistence problems present in daily life is recognized. Furthermore, this study revealed a positive association between the worsening of the physical QoL of riverside dwellers and inadequate FHL, and mental QoL was associated with the socioeconomic and health characteristics of the riverside population.

This study suggests that for every ten riverine people seen in the region’s PHC, eight had limitations and inadequacies in understanding written and numerical information. These results highlighted the marked weaknesses in the FHL of these populations when compared to the urban population in the same PHC scenario in the Brazilian Amazon region. This is a study whose evidence has shown that four out of every ten PHC users may not understand health information [33]. These findings converge to indicate that the rural riverside population has fragile HL functional skills, which require strategies in line with their health needs.

The QoL of people living in rural areas is also affected, as they have logistical difficulties in accessing health services, a high number of elderly people, and low levels of education and family income [34]. A study conducted in Turkey with a population from a semi-urban area, where the basis of subsistence is agriculture, using the SF-36 recorded a physical component score of 70.4 and a mental component score of 64.2 [34]. It presented slightly higher scores than the present study, although they were with a population whose environmental conditions are similar to those of this study, this fact reinforces the social vulnerability to which riverside dwellers are exposed.

In this sense, access to health services in rural areas is a predictor of FHL, as shown in a study carried out in PHC, which concluded that access to health instructions and information contributes to improving HL [35], which is different from the findings of this study, which identified a worsening in the level of FHL of those who lived near the health service. This finding could be characterized as a detection bias, in which the level of FHL is identified based on the greater search for the health service.

In addition, PHC is the gateway to the care network and therefore access to health services, and is responsible for facilitating the development of HL skills, given that organizations and professionals who incorporate HL assumptions into professional practice become facilitators for promoting satisfactory HL since most health promotion information is dispensed at this level of care [35–37].

In addition to these factors, the socioeconomic status of the riverside residents must also be considered. Low levels of schooling are associated with inadequate FHL, in line with other studies that have pointed to schooling as a predictor of HL levels, since it influences everything from the application of basic FHL skills to the conception and interpretation of health information [34]. In addition, people who live in riverside communities carry cultural and traditional traits inherent to their way of life, which are related to extractivist and agricultural activities, knowledge that is passed on through socializing and experiences. Added to this context are the difficulties in accessing school and the lack of financial resources to meet daily needs, resulting in discouragement to continue with their studies [38, 39].

Concerning good physical QoL, an association was confirmed with low schooling, an unusual finding in the scientific literature which more commonly reports a negative statistical association with physical QoL [34, 40, 41], as identified by Amin et al. [42] who recorded low physical QoL scores in individuals with no schooling and a gradual statistically significant increase with levels of education (*p* < 0.05).

It is worth noting that the low level of schooling among the river dwellers associated with better physical QoL could be attributed to the way QoL is measured and to the particular characteristics of this population, who carry out intense physical activity in their work activities, such as fishing, hunting, açaí extraction, and crop cultivation [8, 13]. It should be noted that the population under study had a low level of schooling and is exposed to subsistence activities that require physical effort, causing this association to be underestimated.

These physically demanding work activities may favor self-perception of less difficulty in performing moderate activities, such as climbing stairs, doing less than they would like, facing difficulties at work, and feeling interference from pain. These aspects are all domains investigated in the physical component of the SF-12, used as a measurement tool in this research [25, 26].

In this sense, the results indicate that, despite their low level of schooling, river dwellers can self-report better physical QoL linked to the work context, which can influence their functional capacity and perception of physical health. Self-perception of health involves the recognition of one’s own QoL, permeating culture and value systems according to one’s objectives and life patterns, i.e., subjective to the individual [5].

Still related to their capacity for self-perception, the river dwellers recognize that they experience problems related to education, transport, housing, and violence in their daily lives. These are populations that are at the mercy of various social inequities, such as the lack of basic sanitation and precarious housing, difficulty in accessing Basic and Higher Education, and the need for income to use community or personal river transport. In addition to the violence they face in the territories in different forms, with sexual violence being the most recurrent [43–45].

This scenario, present in riverside communities, is linked to the capacity for social activation to reach advanced levels of HL, such as critical since the individual can observe and analyze a given situation inherent to their reality and use information to exercise control over different life events and situations [46]. Low schooling has been recognized as an obstacle to achieving a better standard of health and life for the population, and there is a need for policies and strategic actions to tackle it. In the global context, the concern of the constituted health authorities can be seen, with the SDGs including the need to eradicate poverty, including improving schooling while observing the inequalities present in the territories [11].

Another socioeconomic characteristic related to HL and QoL is sex. FHL was found to be worse in women, which differs from other studies that have shown a better level of HL in women because they are more participative in health care, are more frequent in health services, and therefore have adopted self-care measures, as well as taking responsibility for looking after their family’s health [47].

However, in addition to making up the majority of the participants in this study (*n* = 209; 67.0%), riverine women are exposed to greater challenges in maintaining their health and to additional socioeconomic factors, since they carried out unpaid activities that have repercussions on their low level of schooling [48]. The influence of factors related to these social vulnerabilities on HL can therefore be seen, as directly impacting on access, understanding, and applicability of health information and, consequently, having repercussions on clinical health outcomes [49].

On the other hand, women had higher physical QoL scores, diverging from evidence suggesting that men have better physical QoL [50–52]. Furthermore, an integrative review of sex differences in the QoL of survivors of hematological malignancies revealed that women have worse physical function [52]. However, caution is needed when making such comparisons, given the particularities of the riverside population of Abaetetuba, a scenario in which women take care of the family and accumulate household activities.

It should be noted that the female sex variable may have been influenced by the Elementary School and limited literacy factors, which also improved the physical component. A survey conducted in China found that elderly women in rural areas had lower levels of education [51]. The improvement in physical QoL among women may be because women were more concerned about their health, and even with difficulties, they attend health services more often, deal with daily domestic activities, and are less sedentary when compared to men. A longitudinal study carried out in Brazil found that low levels of sedentary behavior are associated with an improvement in the physical component of QoL [53].

As for the age groups, in this study, it was possible to observe that there was a significant worsening of the HL with the increase in the age of the participants, a finding that corroborates that shown in other studies when they showed that the higher the age, the lower the HL levels [54]. Thus, this finding can be related to the physiological decline, which interferes with vision and hearing, and cognitive decline associated with increasing age, with the result that performance in tests that measure FHL is compromised [55]. In addition, the level of schooling can influence FHL in older people, as it is common in developing countries for these people to have been deprived of access to schools, which interferes with their ability to read and calculate numbers [56].

In addition, these age factors also influence QoL, since the results obtained here indicated that middle-aged adults had worse physical QoL, in line with research carried out in China in rural and urban areas, which revealed that physical QoL worsens with advancing age and with living in rural areas [51]. It is known that work activities, especially those that require greater physical effort, such as subsistence farming and artisanal fishing, can compromise physical QoL, just as the aging process and lifestyles can cause physical limitations.

Concerning mental QoL, middle-aged and elderly adults had worse MCS. The literature points to widowhood, loss of prospects after retirement, dependence on children, and lack of financial and emotional support from family members as predictors of worse mental health in older people [51, 57]. In addition to these issues, it is known that the impairment of mental QoL in the elderly is influenced by concern about complications arising from illnesses, according to the conclusion of a European study with elderly people diagnosed with any medical condition, which identified low mental QoL scores, especially among individuals with negative feelings and problems concerning the meaning of life [58].

In this sense, mental HL is important among older people since it is related to how they understand and recognize the symptoms of mental disorders, how they deal with self and community stigma, and how they make decisions about seeking out mental health professionals and other sources of information [59].

Regarding physical QoL, it worsened in people with two or more children, which may be due to the demands of care combined with work activities that require physical effort and carrying out household chores. A study that investigated the QoL of caregivers of children and adolescents found that among twenty individuals with more than two children, sixteen had low physical QoL [60].

Regarding economic issues, the results of this study indicated that beneficiaries of the Bolsa Família Program (PBF) had a worsening in their mental QoL, which may be related to the conditions and criteria for enjoying the benefit, as well as to the impacts of the program on improving QoL, which according to studies, are only noticeable in the long term [61].

The *Bolsa Família* Program is a social benefit provided by the Brazilian government for individuals with low or no income, which includes a monthly personal income of R$218,00 (two hundred and eighteen reais), corresponding to 15.4% of the minimum wage in force in November 2024, which totaled R$1,412.00 or US$245.25 (based on the commercial dollar exchange rate of 11/12/2024 of R$5.76) and other social criteria [62]. A study that investigated the QoL of PBF beneficiaries using the WHOQOL-bref revealed an association between better QoL and good/very good health status (67.7%) [63].

However, a review study concluded that income management does not occur in a unique way among Brazilian families, since they are not all equally poor, and those who have the PBF as their only source of income concentrate their spending on food and are late in paying off debts, a fact that generates stress and dissatisfaction with the small amount received [61].

Thus, beneficiaries of the program may exhibit a profile that could potentially worsen mental QoL. It is worth highlighting the importance of income transfer policies for populations in situations of economic vulnerability, such as riverside communities; however, it is necessary to evaluate the program’s premises, intersectoral strategies, and the tiny amount granted to beneficiaries.

In addition, economic issues have an impact on access to health information from the media, especially the rapid expansion of the use of mobile communication devices, which are visibly on the rise around the world and used in different regions and locations. When connected to the internet, they make it possible to disseminate various types of information, such as health-related information. In riverside communities, although there has been an increase in the use of telephone handsets, access to the Internet is still limited, mainly due to the difficulty of geographical access to locations and the lack of investment in implementing network services [64].

Thus, this study showed a relationship between low levels of FHL and the use of cell phones without internet access, which is an important resource for enabling the dissemination of media HL [65]. Thus, it is recognized that in addition to FHL, digital health literacy is also compromised, since it is related to the individual’s interaction with technological resources that enable access to digital information and contribute to coping with limitations and solving individual and collective health problems [66].

Concerning environmental aspects, those who disposed of waste improperly showed an improvement in mental QoL, attributable to the fact that among the forms of inappropriate waste disposal, incineration at home was the practice with the highest proportion. According to the IBGE, in 2022, in rural areas of Brazil, only 31.8% of households had garbage collection, and the main destination for garbage was burning on the property, in 51.2% of households [67].

It is healthy for the riverside population to be engaged in promoting planetary health, since the changes observed in natural systems have contributed to health problems, with vulnerable populations being those with the greatest potential for illness [68, 69]. Therefore, it is necessary to implement public policies aimed at basic sanitation and waste disposal, as well as promoting environmental education for the riverside population.

In this scenario, environmental HL stands out, since it can provide opportunities for knowledge, interpretation, reflection, and attitudes that result in the implementation of measures for sustainable development. The environmentally literate person, in addition to knowing the information, takes a conscious stance to help solve environmental problems [70, 71].

Regarding medication control, it was found that individuals who took their medication correctly had worse mental QoL, which may be related to the dependence on medication shown by people who take it routinely, which in turn are also factors related to worse mental QoL [58]. A contrary fact was identified in a cohort study of elderly Iranians, which found that medication adherence mediates the association between QoL and HL, reinforcing that the negative effects of insomnia and psychological distress include reduced adherence to medication and impaired QoL [72].

Although there is a relationship between HL and QoL, the factors associated with the two are inconsistent, especially among the most disadvantaged groups, as these findings and the literature have pointed to different socioeconomic and environmental characteristics, the use and control of medication and significant problems between HL and QoL. However, as the studies that have addressed these two concepts in a rural context are limited, we decided to sharpen this discussion to contribute to the QoL and HL of this population.

Thus, the riverside population of the Abaetetuba islands is young, with poor basic sanitation and a low level of education, linked to health problems and violence [8]. Thus, these populations that lack the effective implementation of public policies that minimize the challenges imposed on them, making it possible to improve health promotion and disease prevention [43].

### Limitations

This study has limitations regarding the data collection instrument, which includes some self-completion texts that are foreign to the riverside reality and can lead to a bias in understanding and, consequently, in information. Although some strategies were used to minimize this. However, the contribution of this study is that it provides input for local-regional health planning and educational actions in PHC, intending to implement practices that are compatible with users’ levels of FHL. It also favors the proposal of public health policies for the riverside population, for the equitable and resolutive provision of services that support health teaching/education through appropriate strategies that are congruent with people’s way of life.

## Conclusions

Low levels of FHL were identified in the riverside population assisted by the PHC, essentially among females living close to the health service. Some variables related to the conditions of being a river dweller deserve to be highlighted in this context of compromised FHL, i.e. low schooling, aging, and lack of access to the Internet on cell phones. In addition, the impairment of physical QoL among women with low levels of schooling and mental QoL in the over-40s is an important finding for establishing intersectoral policies.

Given the above, it is clear that the level of FHL and QoL are directly related to socio-environmental factors, which are even more challenging among the riverside population since they face problems from different spheres. Thus, recognizing the existing obstacles offers the possibility of seeking a better FHL. To this end, it is necessary to implement specific public policies, since the riverside population has its own unique way of life and social context.

This study identified the relevant aspects related to the health of the riverine population, enabling a reflection on the development of health actions that contribute to minimizing the challenges identified and promoting an improvement in the FHL and QoL of the population.

## Data Availability

The datasets generated during and/or analysed during the current study are available from the corresponding author on the reasonable request.

## Abbreviations

HL: Health Literacy
FHL: Functional Health Literacy
TOFHLA: Test of Functional Health Literacy in Adults
TLS: Health Literacy Test
QoL: Quality of Life
IPQV: Multidimensional Index for Loss of Quality of Life
UHS: Unified Health System
PNAB: National Primary Care Policy
PNSIPCFA: National Policy for the Integral Health of Rural, Forest and Water Populations
SDG: Sustainable Development Goals
RFHT: Riverine Family Health Team
CHA: Community Health Agent
IBGE: Brazilian Institute of Geography and Statistics
SF-12: 12-item Short Form Health Survey
PCS: Physical Component Summary
MCS: Mental Component Summary
SEM: Structural Equation Modeling
SES: Socioeconomic Status
SEX: Sex
AGE: Age Group
SCH: Schooling
SKC: Skin Golor/race
FIL: Number of Children
PLC: Proximity to Health Service
INC: Income
RIG: Defense of Rights
CME: Means of Communication
SWK: Start of Work
DWK: Daily Working Hours
SSB: Social Security Benefit
FAM: Bolsa Família (Family Allowance)
SEW: Sanitary Sewerage
GAR: Garbage Disposal
WAT: Water Supply
MEU: Medication Use
MEC: Medication Control
PBF: Bolsa Família Program
PFE: Problems with Feeding
PED: Problems with Education
PDR: Problems with Drugs
PVI: Problems with Violence
PHO: Problems with Housing
PTR: Problems with Transportation
PFA: Family Problems
PBS: Basic Sanitation Problems
PEC: Economic Problems
PHE: Health Problems
PWK: Work Problems
PLE: Problems with Leisure
PME: Problems with the Media
PLO: Problems with Loneliness
HLI: Inadequate FHL
HLL: Limited FHL
PHC: Primary Health Care
SF-36: 36-item Short Form Health Survey
OLS: Health Literate Organizations
